# Individual differences matter in heritage language bilingual processing: An electroencephalography (EEG) study of grammatical gender

**DOI:** 10.1017/s0272263125101149

**Published:** 2025-09-04

**Authors:** Jiuzhou Hao, Eleonora Rossi, Megan Nakamura, Alicia Luque, Jason Rothman

**Affiliations:** 1 UiT The Arctic University of Norway, Tromsø, Norway; 2 University of Florida, Gainesville, FL, USA; 3 Pennsylvania State University, University Park, PA, USA; 4 Nebrija University, Madrid, Spain; 5 Lancaster University, Lancaster, UK

**Keywords:** grammatical gender agreement, heritage speakers, individual differences, markedness

## Abstract

The present study investigated if/how individual differences in heritage language (HL) experience modulate gender agreement processing among Spanish heritage speakers (HSs). We reanalyzed the data from Luque and colleagues (2023), which reported an aggregate biphasic N400–P600. The present analysis revealed that sensitivity to morphological markedness was positively modulated by HL proficiency and exposure/use. Higher proficiency led to increased P600 across markedness conditions—the typical signature of L1-dominant processing—while increased Spanish exposure/use resulted in increased N400 for Default Errors—a signature attested only in HSs in this domain. Formal instruction led to increased N400 but reduced P600 for Feature Clash Errors. We interpret these results to suggest that the N400 reflects a morphophonological pattern-matching strategy with some HSs relying (more) on this mechanism as Spanish exposure and use increases. Markedness also modulated the relative engagement of pattern-matching (N400) versus automatic grammatical processing (P600), depending on the transparency/saliency of morphophonological patterns.

Heritage speakers (HSs) are early bilinguals who acquire their heritage language (HL) naturalistically as a first language (L1) at home, despite being raised in an environment where the HL is not a dominant language of the greater society (Rothman, 2009). Evidence has demonstrated that HS aggregates perform differently from other native speaker groups raised where the HL is the dominant language of the larger society (Montrul, 2018, 2022; Polinsky, 2006; Polinsky & Scontras, 2020). Performance asymmetries between HSs and L1-dominant users are not unexpected given the manifold differences in language exposure and use that define their realities. Relative to L1-dominant users, HSs typically receive reduced input, have less overall and more restricted opportunities for HL use, and often receive little to no formal training in the HL. And yet, HSs do not merely differ from L1-dominant speakers; they can and do differ from one another to degrees unattested within L1-dominant speaker variation. Taking an approach that shifts away from aggregate comparisons between HSs and L1-dominant counterparts (De Houwer, 2023; Rothman et al., 2023), one is able to describe and unpack the significance of inter-individual differences in HSs.

Recent psycholinguistic research suggests that although HSs and L1-dominant users may show significant quantitative and/or qualitative differences in offline (comprehension and production) measures, they do adopt qualitatively similar processing strategies in online sentence processing tasks (Di Pisa, Kubota, Rothman & Marinis, 2022; Fuchs, 2021, 2022; Fuchs & Zeng, 2024; Hao, Chondrogianni & Sturt, 2024; Ito, Nguyen & Knoeferle, 2024; Jegerski, Keating & VanPatten, 2016; Jegerski, 2018a, b; Luque et al., 2023). However, there is a significant dearth of available online HL processing studies. The present study seeks to bring the above two research gaps—within group, individual differences approach and online processing—together and examine what patterns of an HS’s exposure to and opportunity for use of the HL lead to their individual placement along a continuum for grammatical processing. To do so, we focus on the processing of grammatical gender agreement in Spanish-speaking HSs.

Why gender? Gender has been reported to be a vulnerable domain in HL bilingualism, although such claims are largely made on the basis of HSs showing significant quantitative and/or qualitative differences to L1-dominant users in offline tasks (Gathercole & Thomas, 2005; Kupisch, Akpinar & Stöhr, 2013; Montrul, 2016; Montrul, Foote & Perpiñán, 2008; Polinsky, 2008; Scontras, Polinsky & Fuchs, 2018; Unsworth et al., 2014). Such differences occur both in gender assignment—the lexical representation of a gender value to a particular noun—as well as in agreement—the morphological matching on elements that express a particular gender value (e.g., noun adjective agreement). Given the high frequency, saliency, obligatory nature of Spanish gender agreement and its relatively early acquisition in childhood (e.g., see Mariscal, 2009), differential performance between adult HS and L1-dominant groups is somewhat perplexing. It is not clear what one should conclude from these observable differences, not least since more recent studies adopting online processing methods show that when HSs’ knowledge of gender assignment is controlled for, HSs display qualitatively similar (to L1-dominant users) processing of gender agreement at the group level (Di Pisa et al., 2022, 2024; Fuchs, 2021, 2022; Keating, 2024; Luque et al., 2023).

This qualitative similarity is supported by how HSs have been reported to show sensitivity to morphological *markedness* during online processing of grammatical gender agreement (Di Pisa et al., 2022; Di Pisa, Pereira Soares, Rothman & Marinis 2024; Keating, 2024; Luque et al., 2023) in ways similar to L1-dominant users and highly proficient second language (L2) learners (Alemán Bañón, Fiorentino & Gabriele, 2017; Alemán Bañón & Rothman, 2016; Beatty-Martínez, Bruni, Bajo & Dussias, 2021; López Prego, 2015). Within linguistic theory, the construct of markedness captures the observation that not all elements of a linguistic property have equal status within the system: Unmarked variants are argued to be underspecified relative to fully specified or marked forms (see e.g., Corbett, 2014, for morphology; Mazurkewich, 1985, for syntax; Rice, 2007, for phonology). Although previous research does show that HSs are sensitive to such markedness asymmetries, all existing analyses in the literature pertain to group-level aggregated data, thus only speaking to trends. Our aim is to focus precisely on individual differences, asking if all HSs are (not) equally sensitive to morphological markedness and why (not). Using the data reported in Luque and colleagues (2023), we do this by regressing factors measuring individual HS’s exposure/engagement with morphological markedness on event-related potential (ERP) outcomes.

## Spanish gender system

Spanish has a two-way grammatical gender (henceforth, *gender*) system where nouns are either masculine or feminine. Although neither gender is strictly associated with a particular (morpho-phonological) marker in absolute terms, the trends are overwhelming: 99.8% of nouns ending in -*o* are masculine and 96.3% of nouns ending in -*a* are feminine (Teschner & Russell, 1984). Nouns with these transparent morpho-phonological cues constitute approximately two thirds of all Spanish nouns. Within the remaining one third, other endings such as -*ción* (feminine) and –(*i*)*dad* (*fem*) offer equally strong cues for gender assignment. However, a good portion of nouns in this latter third (e.g., nouns ending in a consonant or the vowel -*e*) do not offer a reliable gender cue and their gender value must be directly learned via exemplars in the input (Harris, 1991; Teschner & Russell, 1984).

While gender is an inherent property of nouns (assignment), all modifying elements within the Determiner Phrase must reflect overt morphosyntactic agreement with the head noun in gender (and number). Otherwise, ungrammaticality arises, e.g., un**a**_-FEM_ cas**a**_-FEM_ roj**a**_-FEM_ versus *un**a**_-FEM_ cas**a**_-FEM_ roj**o**_-MASC_. Given that Spanish highly restricts permissible bare nominals in all argument positions—i.e., most nouns are accompanied by a gender-bearing determiner—and has a relatively transparent agreement paradigm, it is reasonable to claim that the Spanish system provides robust reliability, frequency, and saliency of gender cues in the input.

In terms of markedness, masculine is considered the unmarked form, feminine the marked (Harris, 1991). Evidence for this comes from several observations. For example, inherently genderless elements such as function words (prepositions) and verbs take masculine gender when nominalized referentially, e.g., the preposition *para*“for,” when being modified, could only take a masculine modifier, e.g., *demasiados paras* “too-many-_MASC_ fors-_GENDERLESS._” Similarly, when feminine nouns are conjoined with masculine ones, masculine modifiers are required, e.g., *El gato y la gata están cansados*. “The male cat and the female cat are tired_-MASC-PL._” Additionally, new lexical entries to Spanish typically take masculine gender. Being unmarked, the idea is that the so-called masculine form is un(der)specified for gender as a feature. In other words, masculine is not associated with a true gender value but rather the absence of specific gender. As such, masculine does not only show as the default but is rather “forgiving” relative to the highly specified feminine in agreement and processing terms.

## The role of markedness in grammatical gender processing

The asymmetry between genders has implications for the relative costs associated with processing different types of gender agreement errors. In the case a feminine noun encounters an agreeing element that is masculine, known as a Default Error, the agreement element bears no real featural specification to create a (comparatively) robust conflict with the specified feature of the noun. Conversely, if a masculine noun encounters a feminine agreeing element, also known as a Feature Clash Error, the specified featural configuration of the agreeing element clashes with the un(der) specified feature of the noun, inducing more computational complexity. Although both are errors and should be processed as such, the Default Error is arguably less costly in processing terms and should thus be more difficult to detect and/or require less computational resources to resolve. Conversely, given that there is a salient feature specification clash in Feature Clash Errors, this type of error should be easier to detect but more disruptive to process (McCarthy, 2008).

The effect of markedness in gender processing has indeed been reported in previous studies with L1-dominant users, proficient L2 learners, and HSs (Alemán Bañón, Miller & Rothman, 2017; Alemán Bañón & Rothman, 2016; Beatty-Martínez et al., 2021; Beatty-Martínez & Dussias, 2019; Di Pisa et al., 2022; Keating, 2024; López Prego, 2015; Luque et al., 2023). Starting with L1-dominant users and L2 learners, compared to Default Errors, Feature Clash Errors have been found to be detected earlier in an eye-tracking while reading task (Keating, 2024) and induce longer reading times in a self-paced reading task (López Prego, 2015).

More importantly for the current study, the markedness effect has also been attested using electroencephalography (EEG). Previous studies examining Spanish gender agreement processing (Alemán Bañón & Rothman, 2016; Barber & Carreiras, 2005; Caffarra, Barber, Molinaro & Carreiras, 2017; Caffarra & Barber, 2015; Wicha, Moreno & Kutas, 2004) consistently report that the P600 component is robustly elicited when comparing exemplars with licit and illicit agreement, sometimes accompanied by a Left Anterior Negativity (e.g., Caffarra & Barber, 2015; Caffarra et al., 2017). The P600 is a positive deflection observed between approximately 500 ms and 1000 ms after stimulus onset, with a typical central-posterior distribution. The P600 is usually linked to processes of syntactic reanalysis and repair (Friederici, 2002; Osterhout & Holcomb, 1992; Swaab, Ledoux, Camblin & Boudewyn, 2011). Importantly, Alemán Bañón and colleagues (2016, 2017) showed a robust P600 effect with agreement violations on adjectives from both genders; however, these effects emerged earlier with Feature Clash Errors than with Default Errors. Similarly, Beatty-Martínez et al. (2021) reported a larger P600 effect for Feature Clash Errors than for Default Errors.

Recent evidence suggests that HSs are also sensitive to markedness during online processing. In an eye-tracking while reading task, Keating (2024) found that, among HSs, sensitivity to Feature Clash Errors emerged earlier than that to Default Errors. HSs have also been reported to have longer reading times in a self-paced reading task (Di Pisa et al., 2022, 2024) when processing Feature Clash Errors than when processing Default Errors. In terms of EEG, adopting a moving window analysis, Luque and colleagues (2023) found that for the HSs they studied, gender agreement violations induced not only a P600 but also an N400 effect. The N400 effect is a component usually associated with (semantic) integration (Guajardo & Wicha, 2014; Swaab et al., 2011) and not typically associated with gender processing, at least in studies examining L1-dominant Spanish users, a fact to which we return in greater detail below. Suffice it to highlight for now, importantly Luque and colleagues (2023) found that Feature Clash Errors induced larger N400 and P600 effects compared to Default Errors. Interestingly, the data patterns (the biphasic N400–P600 pattern) from Luque and colleagues lead the authors to suggest that Spanish HSs might exhibit even greater sensitivity to markedness than L1-dominant users. Similar patterns and argumentation can be found in other HL work. For example, Di Pisa et al. (2022) used a self-paced reading task to examine gender agreement processing in Italian L1-dominant users and Italian-German HSs. Results indicated that both groups experienced longer reading times for ungrammatical conditions relative to grammatical ones, but only the HS group’s reading times were significantly modulated by markedness—taking longer to read sentences with Feature Clash Errors than with Default Errors (see also Di Pisa et al., 2024 for similar findings in the role of markedness in the processing of number agreement).

Although preliminary findings suggest that HSs are sensitive to markedness asymmetries during grammatical gender processing at the group level, it remains unclear if individual HSs show differential sensitivity to markedness during gender processing due to their engagement/experience with the HL. In the absence of looking deeper into individuals, can/should we confidently conclude that HSs are more sensitive to markedness as some have recently claimed (e.g., Di Pisa et al., 2022, 2024; Luque et al., 2023)? Is being an HS a sufficient—or even necessary—condition for increased sensitivity to markedness, or is it rather the case that specific HL usage patterns drive such increased sensitivity to markedness for some individuals? The goal of the present study is to investigate if there are some discernible HL engagement characteristics that drive what seem to be a group-level trend toward greater sensitivity to markedness in grammatical gender processing, and if so, to unpack and consider why.

## Individual differences in (HL) sentence processing

An interesting finding from Luque and colleagues (2023) that suggests an individual differences approach could be particularly revealing is the observed biphasic N400–P600 component (modulated by markedness) at the group level. While the typical P600 signature was present, recall that the N400 is not a signature associated—at the group level—with syntactic gender processing, at least in sentential contexts. The authors offered two, not mutually exclusive, postulations as to why the N400 was also observed in their data along with the P600. First, they pointed to the possibility that individual differences in the preferred processing “route” might have contributed to the observed N400–P600 biphasic pattern (an illusion of group-level averaging). The authors suggested—but did not actually test—the possibility that (some) individual HSs might have been more N400 than P600 dominant in how they process syntactic anomalies and the ratio in their particular cohort was such that both signatures survived aggregated averaging. If this were the case, then there is no bona fide N400/P600 per se but rather a semblance of one. Indeed, such a pattern has been observed both between and within subjects in both L1 and L2 populations (Grey, 2023; Kim, Oines & Miyake, 2018; Tanner, 2019; Tanner, Inoue & Osterhout, 2014; Tanner & Van Hell, 2014). However, while this individual difference pattern has been attested, as in the aforementioned studies, differently from Luque and colleagues, it is not the case that both signatures have survived group averaging. Rather, these datasets documenting N400-dominant and P600-dominant individuals co-existing within groups that otherwise average to show either a P600 or N400 highlight the caution one must take with averaging in general and interpreting what a particular ERP signature means. Second, and more probably according to the authors, it was postulated that the N400 could reflect HSs’ enhanced sensitivity to overt morphology. Here, HSs made use of overt morphology to engage in pattern-matching/integration for grammatical gender agreement processing. As such, according to the second postulation, HSs engaged in both pattern-matching (N400) and automatic grammatical processing (P600) at the same time, at least at the group level. It is, therefore, critical to understand what individual factors modulate or otherwise impact the elicitation of distinct ERP signatures during Spanish gender processing—what individual differences factors modulate the relative engagement in one “route” or another or both.

Given the two postulations proposed by Luque and colleagues (2023), the present study examines three individual differences factors based on previous (HL) processing literature, i.e., HL proficiency, HL formal instruction, and overall exposure to and use of the HL. Starting with the role of proficiency, previous L2 processing studies using EEG have found that for the processing of grammatical (dis)agreement, while high(er) proficiency learners showed P600 components, low(er) proficiency learners showed N400 components (e.g., Alemán Bañón et al., 2018; Grey, 2023; Morgan-Short, Sanz, Steinhauer & Ullman, 2010; Osterhout, McLaughlin, Pitkänen, Frenck-Mestre & Molinaro, 2006). More recently, Hao, Kubota, et al. (2024) showed that HSs with HL formal instruction are more likely to engage in pattern-matching during sentence processing over and above processing using grammatical cue(s). The authors argued that HL formal instruction, a proxy for formal literacy practice, may lead to enhanced metalinguistic awareness in the HL that favors pattern-matching as a processing strategy. Hao, Kubota, et al. (2024) also empirically demonstrated the role of overall exposure and use of the HL for HL sentence processing—more HL exposure and use are associated with more efficient HL processing (see also Bayram, Pisa, Rothman & Slabakova, 2021; Montrul, 2022; Paradis, 2023; Prystauka, HaoCabrera Perez & Rothman, 2024, for discussion).

## The present study

Focusing on gender agreement with morphological markedness manipulations among Spanish HSs, we revisit parts of the aggregated ERP dataset reported on in Luque and colleagues (2023). The advantage of using this dataset is severalfold: (a) it includes a comprehensive language background questionnaire and a proficiency measure and (b) it adopted a moving window analysis, differently from using predefined time windows typically done in ERP studies. The former, (a), provides information to use in regression analyses to investigate what, if any, language exposure/use factors are predicative for individual differences. On the basis of the latter, (b), an N400 was revealed that otherwise could have gone unnoticed, given that the time-window would not necessarily have been looked at otherwise (the N400 does not typically show up in Spanish L1-dominant users’ gender processing). This N400 could be a novel signature specifically of HL processing (in this domain), as suggested in Luque and colleagues (2023). However, one of our goals is to understand if it applies always or equally for all HSs, which is to say how generalizable is itas a marker of HL processing in this domain? More specifically, we ask, Which (and how do) individual level bilingual language experience factors (HL proficiency, HL formal instruction, and HL exposure and use) modulate ERP signatures? If so, do they do so differentially for marked and unmarked agreement errors?

There are two logically possible outcomes, i.e., individual HSs either differ from each other or not. We predict an effect of individual differences in the processing of gender agreement as a function of their respective experience/engagement with Spanish, e.g., exposure to and usage of Spanish, proficiency of Spanish, and formal training in Spanish (e.g., Grey, 2023; Hao, Kubota, et al., 2024; Prystauka et al., 2024). In particular, if the N400 and the P600 during grammatical violation processing index only differential engagement for processing routes (postulation one of Luque and colleagues), we expect that with the increase of HL proficiency there would be a decrease of N400 but an increase in P600 (e.g., Grey, 2023). If the N400 also reflects, to some degree, sensitivity to morphology and pattern-matching (postulation two of Luque and colleagues), HL formal instruction may lead to enhanced N400 (Hao, Kubota, et al., 2024). As for the effect of overall HL exposure and use, if it modulates HL sentence processing efficiency (Hao, Kubota, et al., 2024; Prystauka et al., 2024), increased HL exposure and use is expected to lead to increased ERP components.

To the best of our knowledge, no previous HL studies using EEG have directly examined if and how individual differences are modulated at all, much less including with markedness. Thus, it is unclear whether individual-level bilingual language experience factors matter to different degrees for brain-based signatures of syntactic processing in general and specifically when markedness is considered.

## Methodology

As reported in Luque and colleagues (2023), the dataset was collected in two sessions, a pre-screening and an in-lab experimental session. During the pre-screening, consent, language background information, and information on general health and handedness were collected. During the in-lab session, a lexical decision task in Spanish and a Spanish gender assignment task (including the full set of nouns used in the main EEG experiment) were completed before the EEG testing. For the EEG testing, to minimize carry-over effect from the lexical decision task, a resting-state EEG and a Flanker task were administered before the grammaticality judgment task with EEG (the main experiment). Upon completion of the study, participants were debriefed and compensated with either course credit or a $40 gift card. Institutional ethics approval was granted prior to the study.

### Participants

A total of 44 Spanish–English HSs participated in the study. For the final ERP analyses in the original study, Luque and colleagues (2023) included 40 participants after excluding four participants due to incomplete dataset and data quality issues. In the present study, we further excluded one participant who did not complete the language background questionnaire, leaving a final sample of 39 participants (29 female, mean age = 20 years; standard deviation [*SD*] = 1.55). All HSs were exposed to Spanish from birth at home and to the societal dominant language, English, either simultaneously or as an early L2 during childhood (mean English first-exposure = 3.7 years of age). Additionally, four participants reported also being HSs of Brazilian Portuguese. At the time of testing, all participants were enrolled as undergraduates at a large Southeastern university in the United States. Participant eligibility was determined via the pre-screening questionnaire, requiring that they (a) had been exposed to Spanish naturalistically at home; (b) had normal or corrected-to-normal vision and hearing, were right-handed; and (c) had no history of neurological or learning disorders.

## Language background

The Language History Questionnaire (version 3; LHQ3; Li, Zhang, Yu & Zhao, 2020) was used to collect participants’ language background information. The LexTALE Spanish version (Izura, Cuetos & Brysbaert, 2014) was administered as an objective proficiency measure. For the 39 participants, the LexTale score had a mean of 64.7 (*SD* = 8.91). In addition to the LexTale score, two language background variables were extracted from the LHQ3. First, based on self-report, we categorized HSs into two types, i.e., those who had experience with formal Spanish instruction, e.g., classroom, and those who had none. This constitutes the binary categorical variable, i.e., *Formal Instruction*, that was used in the modelling of the present study. Of the 39 participants, 18 had *Formal Instruction*.

Second, we calculated a *Ratio of Exposure and Use* score, which yielded a mean score of .32 (*SD* = .23). Specifically, we calculated the *Exposure and Use* score respectively for English and Spanish and took the ratio between the scores as the *Ratio of Exposure and Use* score. Similar to the *Ratio of Dominance* score, a standardized score provided by the LHQ calculator, the *Ratio of Exposure and Use* score, puts all participants onto the same scale in an effort to circumvent the reality that some participants may be more/less liberal when estimating their language use. To calculate the *Exposure and Use* score for each language, the formula $\sum_{j=\{Reading,Writing,Speaking,Listening\}}\omega_{j}\left(\frac{H_{ij}}{K}\right)$ was used, where $H_{\text{ij}}$ stands for the total estimated hours per day one spent on the j^th^ linguistic aspect of the i^th^ language; K is set to be 16 as a constant scaling factor and $\omega_{j}$ to .25 as a weight assigned to each component. While the spirit of scoring this way is in line with the *Language Dominance* score from the LHQ calculator, it deviates from it by not including self-rated proficiency for reasons we discuss below.

One benefit of using questionnaires like the LHQ is that they allow for theory-driven flexibility with respect to the formulas used to derive composite scores. This is crucial since some factors have differential weightings depending on the type of bi-/multilingual in general (e.g., early versus late acquired bilingualism) and/or for groupings of any given bilingual aggregate. In other words, this addresses the fact that not all the same factors have equal impact/relevance in all circumstances by allowing for the constant meta-data collected via the questionnaire to be used differentially, driven by contextual factors fitting the bespoke needs of particular bi-/multilingual types and/or specific, real-world groupings of them. In the case of HSs, at least for the present sample, we would argue that the standard LHQ formulations of *Dominance Ratio* and *Language Immersion* are not ideal measures, which is why we instead calculated the *Ratio of Exposure and Use* score from the LHQ data, as described above. As can be appreciated in the LHQ formula for calculating dominance, Dominance = ∑j={Reading,Writing,Speaking,Listening}ωj(12(Pij7)+(12(HijK))), it uses self-reported proficiency and weighs it heavily (50%). As the present study collects a more objective measure of proficiency separately and runs it in the modelling to account for individual differences, using the Ratio of Dominance score would constitute double dipping, leading to potential underestimation of an effect of proficiency. It is worth noting here, nevertheless, that our *Ratio of Exposure and Use* score is highly correlated with the *Ratio of Dominance* score (correlation coefficient = .78).

As can be further appreciated in its formula, Immersion = $\frac{1}{2}\sum_{j=\{Reading,Writing,Speaking,Listening\}}\omega_{j}\left(\frac{Age-AoA_{ij}}{Age}+\frac{YoU_{i}}{Age}\right)$, the LHQ Language Immersion score makes use of the age at which one started using a given language in different modalities. This assumes that one has approximately the same exposure and use pattern of the languages across the lifespan, or at least, variation and change in exposure and use are constant across participants. This, however, is not a fair assumption to make for HSs, generally and for those in the present study in particular. Moreover, the current HS group is relatively homogenous in terms of age and age onset of Spanish and/or English, critical information factored into the calculation of the *Language Immersion* score (mean = .57, *SD* =.04). This lack of variability also has implications from a statistical perspective, i.e., restricted variability in the predictor(s) might lead to less variance explained by statistical models. In contrast, our participants differ more drastically in their daily use of Spanish and English, the primary information used to calculate the *Ratio of Exposure and Use* score (mean = .32, *SD* = .23). It is in the domains of opportunities for dual language engagement brought about by variation in usage patterns that our hypotheses expect to find correlational significance. To be conservative to start, we ran separate (maximal) models and found the ones including the *Ratio of Exposure and Use* score to have higher adjusted *R^2^* values than the ones including the *Language Immersion* score. We are confident as a result that the *Ratio of Exposure and Use* score indexes more/less exposure and use of Spanish (relative to English), thus constituting the optimal score for our purposes.

## Grammaticality judgment task

The main experimental task was an EEG grammaticality judgment task. Participants were asked to read sentences in a rapid serial visual presentation paradigm, i.e., sentences were presented one word at a time in the center of the screen. At the end of each sentence, participants were instructed to judge the grammaticality of the sentence via a button-press using an external keyboard.

Grammaticality was manipulated in terms of gender agreement between target nouns (used in a gender assignment task) and the adjacent postnominal adjectives, i.e., the adjective either agreed (Grammatical) or disagreed (Ungrammatical) with the target noun in gender. To examine the effect of Markedness, we manipulated the gender specification of the target noun (Masculine and Feminine). This 2 × 2 design (Grammaticality by Markedness) led to four experimental conditions: Grammatical Masculine Noun (with masculine adjectival agreement), Grammatical Feminine Noun (with feminine adjectival agreement), Ungrammatical Masculine Noun (with feminine adjectival agreement, corresponding to Feature Clash Errors), and Ungrammatical Feminine Noun (with masculine adjectival agreement, corresponding to Default Errors) (see Table 1, for examples).

A total of 900 sentences were created and evenly divided into three lists such that each list consisted of different experimental items. Within each list there were 40 items per condition—160 experimental trials in total for gender agreement, to achieve the recommendation by Molinaro, Barber & Carreiras. (2011). For experimental trials, all target nouns were inanimate such that they had grammatical gender but not semantic or natural gender. Additionally, half of the nouns had transparent endings (masculine - *o* and feminine -*a*) while the other half had opaque endings (-*e* or consonant). Also unique to each list, 80 sentences that were part of another study where gender agreement is not the focus were included. This created 720 unique sentences in total that were not shared across lists. The remaining 180 sentences were shared across lists, including 150 filler items and 30 ungrammatical sentences where gender agreement was violated between the determiner and the noun, e.g., *Mariano fotografió unafem tornadomasc peligrosomasc (*Mariano photographed afem dangerousmasc tornadomasc). The latter was to ensure that participants did not rely on the gender information encoded in determiners as a (additional) cue because gender-bearing determiners preceding nouns are obligatory in Spanish. In total, each list consisted of 420 sentences. A total of six blocks (70 items each) were created. All sentences within blocks were randomized. Each trial started with a 500 ms fixation cross, followed by a 150 ms interstimulus interval. Each word appeared in the middle of the screen for 300 ms followed by a 150 ms interstimulus interval for all sentence items except for the last one. The next trial began following a response. The task took approximately 50 min to complete.

## EEG recording and pre-processing

Continuous EEG was recorded using an array of 32 Ag/AgCl scalp active electrodes (BrainVision, Brain Products GmbH, Gilching, Germany) organized in accordance with the 10–20 system. For online referencing and later re-referencing, two electrodes were respectively placed on the right and the left mastoid. Impedance was maintained at <10 kΩ. Additionally, two sets of bipolar electrooculogram electrodes were placed above and below the left eye and on the right and left canthi to respectively measure vertical and horizontal eye-movements. A BrainVision actiCHamp amplifier with a 24-bit analog to digital conversion was used to amplify the signal that was continuously recorded at a 1,000 Hz sampling rate without online filters. All data were pre-processed offline using Brain Vision Analyzer (version 2.2; Brain Products GmbH, Gilching, Germany). EEG data were re-referenced to the average of both mastoids and filtered using a .1–30 Hz IIR Butterworth filter with a 12 dB slope. Independent components analysis (ICA) was used to identify and remove vertical and horizontal eye movements. After ICA, the data were subjected to a final inspection, using a semi-automatic filtering mode followed by visual confirmation. The continuous EEG signal was then segmented into epochs relative to the adjectives (−200 ms to 950 ms) and baseline corrected (−200 ms to 0 ms). All stimuli, data, and analyses scripts can be found on the Open Science Framework (OSF) page (see the Data Availability Statement for the link).

## ERP individual differences data extraction

Traditionally, three ERP measures have been adopted to capture individual differences, e.g., Response Magnitude Index, Response Dominance Index, and raw amplitude (Grey, 2023; Kim et al., 2018; Tanner, 2019; Tanner et al., 2014; Tanner & Van Hell, 2014). These approaches, however, are not without some limitation given that they all rely on the averaging of amplitude across items, time, and electrodes. Such averaging complicates comparisons across participants (and conditions) when outliers and the signal-to-noise ratio differ across participants (and conditions). Additionally, such approaches typically use arbitrarily predefined time window that varies studies to studies, which may also ignore potential individual differences in latency. To address these issues, an alternative method has been proposed by Meulman, Sprenger, Schmid & Wieling (2023), which the current study adopts with minor adaptations, as described below. More specifically, Meulman and colleagues used Generalized Additive Models (GAMs), a non-parametric regression technique, to smooth ERP data throughout the whole-time trajectory per participant and condition. Additionally, item-level information is retained and included as a random effect, accounting for the variability in ERP responses across different trials. Several indicators for individual differences can be extracted from these GAM-smoothed difference waveforms (between Ungrammatical and Grammatical condition), instead of from the raw, underlying (noisy) EEG recording.

Of particular interest for the present study and following Meulman and colleagues’ recommendation, we adopted the Modeled Peak Latency (MPL) and Normalized Magnitude Peak (NMP) as variables of interest, respectively, for timing (latency) of response and robustness of response (response stability) of the N400 and P600 components. It is worth noting that the NMP not only reflects the amplitude of the ERP responses but also incorporates the variability of the signal, making it a robust measure of response consistency. To extract NMPs and MPLs, we ran GAMs in R (R Core Team, 2018) for each participant separately for the N400 effect and the P600 and for the Masculine Noun Conditions and the Feminine Noun Conditions. Following Meulman et al.’s (2023) recommendation, we did so separately for each electrode of interest. More specifically, we included electrodes that are typically used in the (bilingual) sentence processing literature (see Kaan, Dai & Xu, 2023), i.e., “FC1,” “FC2,” “C3,” “Cz,” “C4,” “CP1,”“CP2,” “CP5,” “CP6,” “Pz,” “P3,” and “P4.” Additionally, we separately extracted these indicators for the dataset with all trials included and the dataset excluding trials where the participant assigned the incorrect gender value in the gender assignment task. We only report analyses of the latter in the main text below as the results from both overlap (analysis on the full dataset is available in the OSF). Differently from Meulman et al. (2023), instead of extracting data throughout the whole-time interval for both N400 and P600, we restricted our time window for extractions to 200 ms to 550 ms and 400 ms to 950 ms, respectively. These time windows are longer than typical time windows for N400 and P600. This enables us to not only account for individual variations in ERP component latencies but also avoid identifying several peaks or a lack of a true peak and identifying a different negative/positive component other than N400/P600—a limitation of Meulman et al.’s (2023) approach.

**Figure 1.**
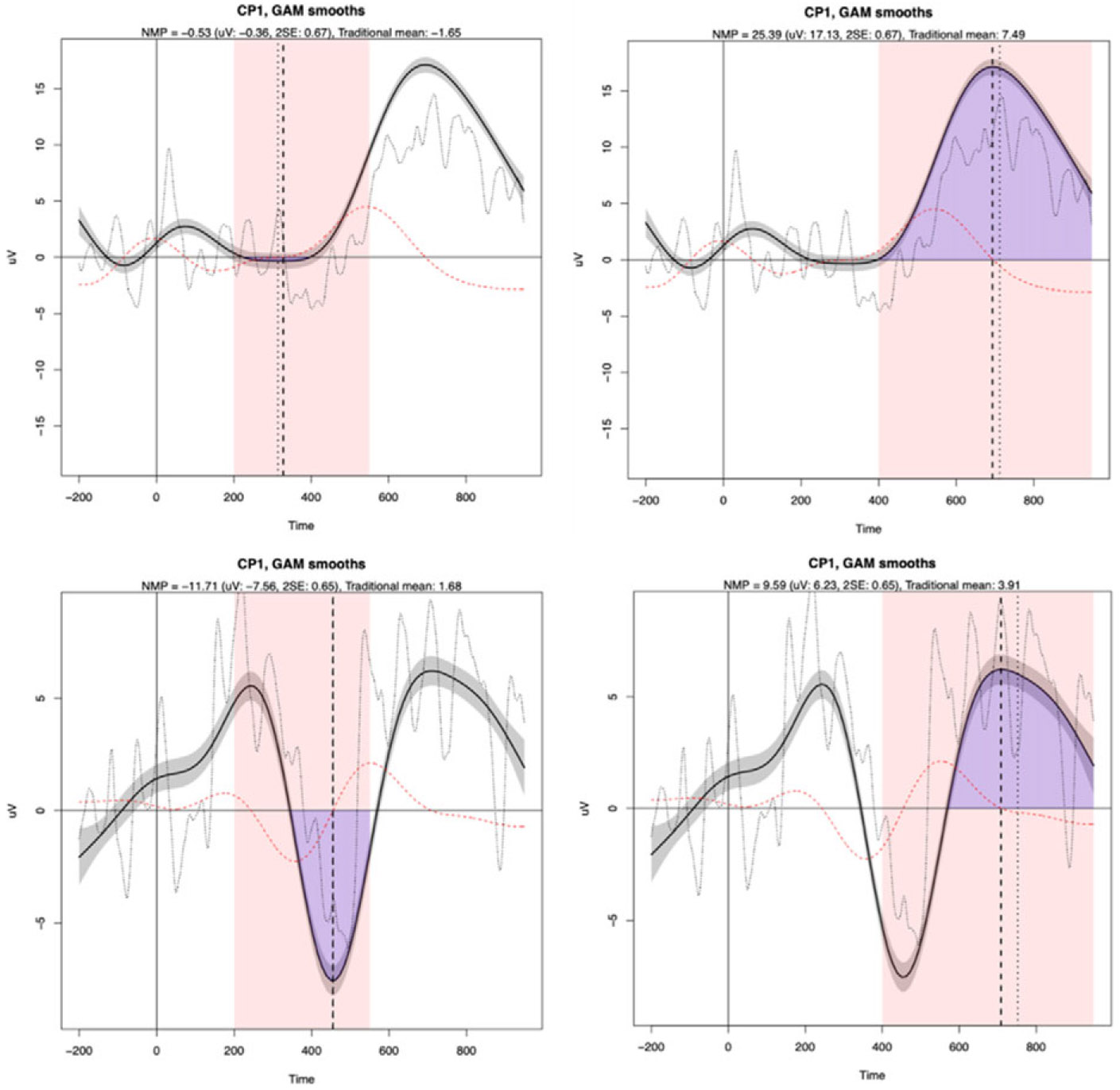
Illustration of GAM-based extraction for one participant at the CP1 electrode site for Feature Clash Error (top) and Default Error (bottom) in the N400 search window (left) and P600 search window (right).

Figure 1 demonstrates the extraction results for Feature Clash Error (top) and Default Error (bottom) in the N400 search window (left) and P600 search window (right) for the same participant at the CP1 electrode site. We refer the readers to Meulman et al. (2023) for the mathematics behind the extraction and the more detailed interpretation of the figures. Here, for our current purpose, the red shaded areas are the time windows in which the extractions were based on, the purple area represents the identified modeled area (the height of which divided by 1.96 times the standard error (*SE*) constitutes the Normalized Modeled Peak and the long-dotted line indicates the MPL.

## Individual differences analysis

For statistical analyses, we used the above extracted values as dependent variables. We adopted linear mixed effect regressions, along with pairwise comparisons with Bonferroni corrections and relevelled models as post hoc analyses, to statistically examine the role of HL proficiency (LexTale), HL exposure and use (Ratio of Exposure), and Formal Instruction and their respective interactions with Markedness/Error Type. We ran models including Markedness/Error Type interacting, respectively, with LexTale score, Ratio of Exposure score, and Instruction, i.e., R syntax: ~ Error_sum * (LexTale_O_C + Ratio_Exp_Spanish_C + Instruction_sum), as fixed effects. We included maximal by-participant and by-electrode random effects where possible. However, we simplified the random effect structure when convergence was not achieved. All categorical fixed effect variables were sum-coded and numerical variables were centered around the mean. Prior to statistical modeling, we examined the correlations among all numeric individual difference variables and conducted linear regressions between the categorical individual difference variable (Instruction) and all numeric variables. The results indicated that none of the individual difference variables were significantly correlated with each other (all *p* values > .05). Additionally, we calculated the Variance Inflation Factor (VIF) for each model to confirm that multicollinearity was not a concern (all VIFs < 2).

## N400 as the effect of interest

### Modeled peak latency

The analyses included a significant effect of Error Type such that N400 MPL is earlier for Feature Clash Errors than for Default Errors (estimate = 9.83, *SE* = 3.36, CI [3.23, 16.42], *t* = 2.93, *p* < .01).

### Normalized magnitude peak

**Figure 2.**
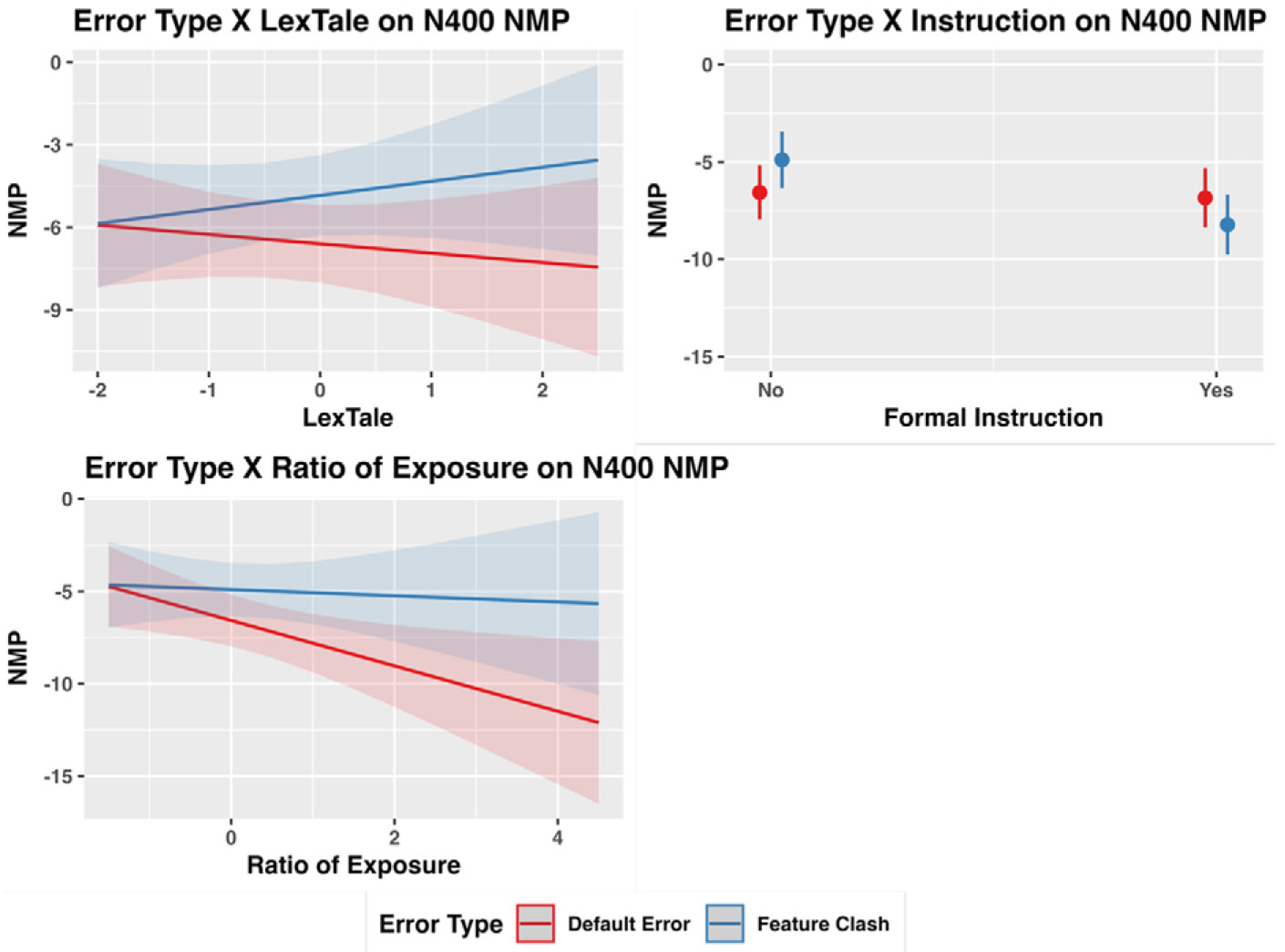
Significant interactions between Error Type and LexTale (top left), Ratio of Exposure (bottom left), and Formal Instruction (top right) for N400 NMP.

Note that as N400 effects are negative values, a smaller (in mathematical terms) N400 NMP indexes a larger N400 effect. The regression model included three significant interaction terms between Error Type and (a) LexTale (estimate = −.43, *SE* = .20, CI [−.82, −.03], *t* = −2.13, *p* = .03), (b) Ratio of Exposure (estimate = −.53, *SE* = .21, CI [−.92, −.14], *t* = −2.70, *p* < .01), and (c) Instruction (estimate = −.76, *SE* = .18, CI [−1.12, −.41], *t* = −4.27, *p* < .001). Figure 2 (top left) illustrates the interaction term between Error Type and LexTale. Post hoc analyses revealed that the Error Type effect is larger in those with higher LexTale than those with lower LexTale. However, LexTale was not a simple effect modulating NMP for either Default Error (estimate = −.34, *SE* = .53, *t* = −.64, *p* = .53) or Feature Clash Error (estimate = .51, *SE* = .57, *t* = .90, *p* = .37). Figure 2 (bottom left) illustrates the interaction term between Error Type and Exposure and Use of Spanish. Post hoc analyses revealed that the Error Type effect is larger in those with more Spanish exposure and use than those with less. Additionally, Exposure and Use of Spanish negatively modulated NMP values, and thus, positively modulated N400 effects, for Default Errors (estimate = −1.20, *SE* = .50, *t* = −2.46, *p* = .02) but not for Feature Clash Error (estimate = −.17, *SE* = .56, *t* = −.30, *p* = .76). Lastly, the interaction term between Error Type and Instruction (Figure 2, top right) was driven by the fact that Feature Clash Errors induced smaller NMP values (larger N400 effects) for those with instruction than those without instruction (estimate = −3.33, *SE* = 1.10, *t* = −3.03, *p* < .01), which was not found for Default Errors (estimate = .28, *SE* = 1.08, *t* =.26, *p* = .80).

## P600 as the effect of interest

### Modeled peak latency

No significant effect was found for P600 MPL (all *p* values > .5).

### Normalized magnitude peak

**Figure 3.**
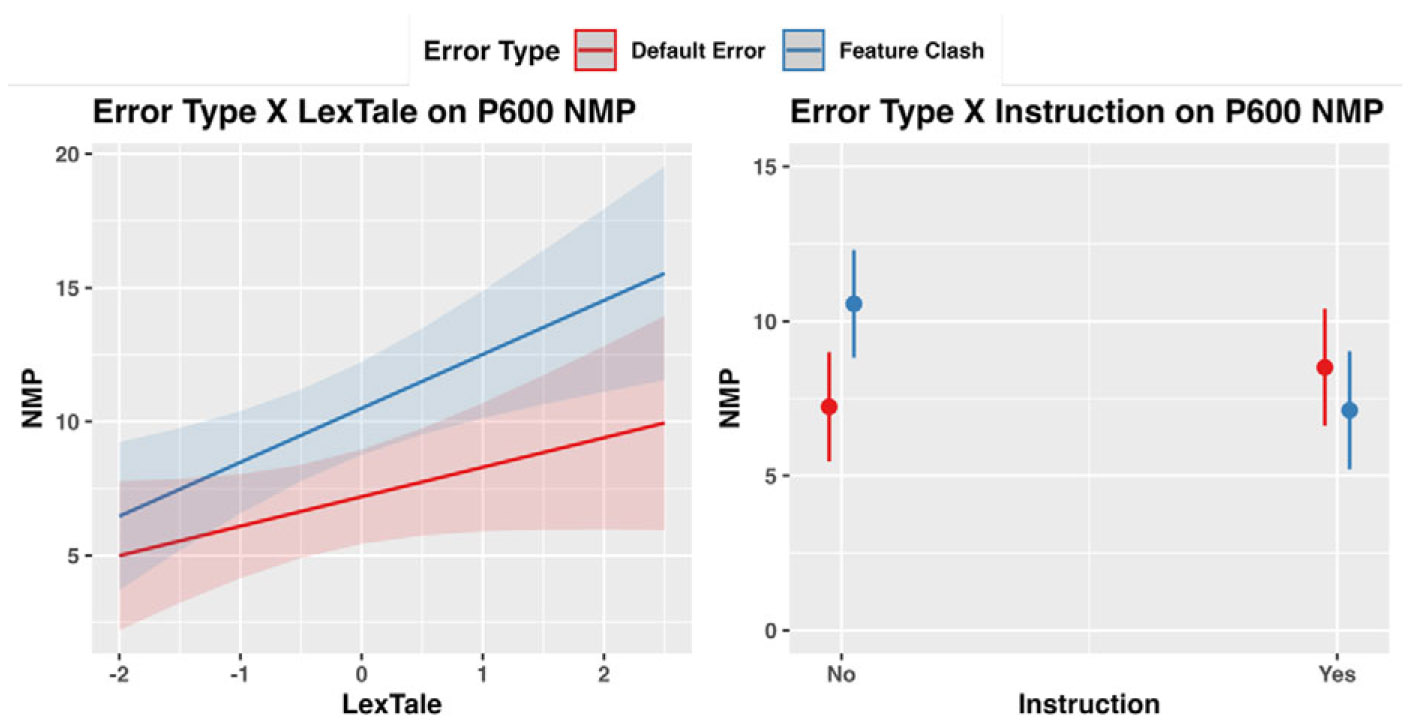
Significant interactions between Error Type and LexTale (left) and Formal Instruction (right) for P600 NMP.

The regression model identified a significant main effect of Error Type (estimate = −.47, *SE* = .19, CI [−.85, −.10], *t* = −2.47, *p* = .01) and LexTale (estimate = 1.56, *SE* = .63, CI [.33, 2.79], *t* = 2.48, *p* = .01) as well as the interaction term between Error Type and LexTale (estimate = −.46, *SE* = .19, CI [−.83, −.08], t = −2.39, *p* = .02) and between Error Type and Instruction (estimate = −1.18, *SE* = .20, CI [−1.57, −.78], *t* = −5.86, *p* < .001) but not between Error Type and Ratio of Exposure (estimate = .12, *SE* = .18, CI [−.23, .47], *t* = .69, *p* = .49). Together with the post hoc analyses (see Figure 3 for visualization), these results suggest that the Error Type effect is larger in those with higher LexTale than those with lower LexTale. Additionally, LexTale positively modulated the NMP for both Default Error and Feature Clash Error (main effect of LexTale). The interaction between Error Type and Instruction was driven by the fact that Feature Clash Error induced smaller NMP for those with instruction than those without instruction (estimate = −3.44, *SE* = 1.35, *t* = −2.55, *p* = .02), which was not found for Default Error (estimate = 1.27, *SE* = 1.35, *t* =.94, *p* = .35).

## Discussion

The present study had the primary goal of investigating how differences in HL experience/engagement may correspond to individual gender agreement processing. We further explored whether markedness played a modulatory role at the individual level, independently or in combination with other individual differences factors. Specifically, we reanalyzed Luque and colleagues’ (2023) ERP data from which a bi-phasic N400–P600 pattern at the group level was reported. The present study regressed HL exposure and experience factors in interaction with morphological markedness to GAM-based ERP responses (Meulman et al., 2023) for both N400 and P600. This allows us to move beyond aggregated trends to address if/how individual level bilingual language experience factors modulate ERP signatures, potentially differentially so, for marked and unmarked gender agreement errors.

Following previous research (e.g., Grey, 2023; Hao, Kubota, et al., 2024; Prystauka et al., 2024), three individual difference factors in terms of participant’s experience with Spanish were extracted and analyzed, i.e., Spanish proficiency as measured by the LexTALE (LexTale score), whether the participant had formal instruction in Spanish, and the ratio of exposure and use of Spanish relative to English (Ratio of Exposure score). The results showed that these three factors all significantly modulated the ERP signatures, albeit in different ways and differentially interacted with markedness.

Starting from the effect of LexTale/proficiency, a main effect of LexTale was attested for P600 NMP, importantly, for both error types—a larger P600 NMP was found for participants with higher proficiency. While acknowledging that HSs are native speakers of Spanish, it is interesting that this effect is in line with findings from other, non-native bilinguals, namely within the L2 literature where the P600 effect is often observed in advanced L2 learners during grammatical processing and modulated by proficiency (Alemán Bañón et al., 2018; Bice & Kroll, 2021; McLaughlin et al., 2010; Rossi, Kroll & Dussias, 2014). For N400 NMP, however, such a main effect of proficiency was not significant nor was its simple effect for each error type. More importantly, there was no significant negative effect of proficiency on N400 effects (or a positive relationship between LexTale and N400 NMP values). The observed positive main effect of proficiency on P600 in the absence of a negative effect on N400 is especially telling. To the extent that individual differences in processing route, i.e., N400 versus P600, are (partially) modulated by proficiency (e.g., Grey, 2023), and that N400–P600 routes are essentially mutually exclusive during grammatical processing, it would be expected that proficiency will influence N400 and P600 at the same time but in the opposite direction, i.e., an increase in P600 would entail a decrease in N400. The present effect of proficiency, therefore, indicates that both N400 and P600 routes of processing were employed by at least some of the same participants. This observation aligns with the second postulation put forward by Luque and colleagues (2023), namely that the N400 is indexing something other than grammatical processing per se in the sense of syntactic repair. It is worth mentioning here that although we postulated that both N400 and P600 routes of processing may be employed by the same participant(s), it does not mean all participants did so or did so to the same extent. What is important here is that the current findings cannot be accommodated if one takes the position that individuals either take a so-called N400 or P600 route for the same, particular function such as syntactic integration/repair. The results, thus, could be interpreted as lending support to Luque and colleagues’ (2023) second postulation whereby they argued the present N400 is indexing increased sensitivity to morphology in (some) HSs: increased HL proficiency is associated with enhanced sensitivity to the morphological markedness effect during processing, regardless of the processing route.

Turning to the effects of Ratio of Exposure (Spanish over English), we observed a significant interaction term between Error Type and Exposure and Use for the N400 NMP such that participants with more Spanish exposure and use showed larger markedness effects. Recall that the N400 effect among HSs has been interpreted as an indication of their increased sensitivity to morphology (Luque et al., 2023). If on the right track, it should follow naturally that more exposure and use of the HL would have manifested as observed. Additionally, we found a simple effect of Ratio of Exposure on N400 NMP for Default Errors only—N400 NMP for Default Errors decreased (an increase in N400 effects) with the increase of Spanish exposure and use, but N400 NMP for Feature Clash Errors was not modulated by Spanish exposure and use. The reason why increased Spanish exposure selectively modulates N400 effects for Default Errors but not Feature Clash Errors is not clear. One possible explanation is that the feminine determiner una provides a highly transparent morphophonological cue (-*a*), facilitating pattern-matching with adjective endings. In contrast, the masculine determiner *un* lacks such a salient feature, making pattern-matching more effortful. As a result, processing Feature Clash Errors may require morphosyntactic integration to a larger degree, making the use of surface-level cues less sensitive to exposure and use effects in processing this type of errors. This aligns with previous research showing that HSs benefit from the presence of transparent morphological and morphosyntactic cues (e.g., Di Pisa et al., 2024; Fuchs & Sekerina, 2025; Hao, Chondrogianni, et al., 2024). Future research could further investigate this by manipulating grammaticality through (dis)agreement between determiners and nouns/adjectives in Spanish. Alternatively, examining a gendered language without gendered articles (e.g., Polish or Russian) could provide additional insight into noun-adjective agreement processing.

Lastly, for the effect of Spanish Formal Instruction, Feature Clash Errors but not Default Errors induced larger N400 effects and, at the same time, smaller P600 effects for those with instruction compared to those without. It is not immediately clear why formal instruction would have such an effect. One idea worth considering is that explicit instruction has placed emphasis on less transparent patterns, on the one hand, and has the effect of nullifying the role markedness might otherwise have played in grammatical processing and syntactic repair, on the other. More specifically, as discussed above, Default Errors are easier to process through pattern-matching given the transparency of gender cues encoded on the feminine determiner, leading to larger N400 effects in individuals without formal instruction. In contrast, in instructional contexts, explicit emphasis may be placed on less transparent patterns—un as a masculine determiner—heightening sensitivity to this otherwise less salient cue (see also Hao, Kubota, et al., 2024). Additionally, instruction in HS contexts, often although not exclusively, parallels much more closely the explicit type that L2 learners receive as opposed to the type of language (arts) instruction L1-dominant users get. At the same time, unlike L2 learners, HSs already have a naturalistically acquired grammatical gender system—like L1-dominant users do—before receiving explicit training. As such, it is possible that such explicit instruction on agreement serves to reduce, if not flip the effects one might otherwise expect without it being based on markedness consideration alone. Especially when one considers the fact that, there being no markedness per se implicated in the Default Error context, instruction does not have the same opportunity to manifest a difference. If on the right track, this would mean, then, that explicit instruction can have a nullifying effect on at least the processing reflexes of preexisting grammatical representations, a hypothesis best tested through longitudinal and/or developmental studies.

## Conclusion

The present study had the goal of unpacking individual differences in gender agreement processing among Spanish HSs. Our results revealed that higher HL proficiency led to increased P600 across markedness conditions while greater Spanish exposure and use resulted in increased N400 for Default Errors. Formal instruction was associated with an increased N400 but a reduced P600 for Feature Clash Errors. Additionally, we found that sensitivity to morphological markedness was positively modulated by HL proficiency and exposure/use. These findings suggest that the biphasic N400–P600 pattern at the group level is more likely to reflect individual differences where the N400 and the P600 index distinct processing mechanisms differentially engaged by individual HSs depending on HL experience and markedness. More specifically, the N400 may reflect a morphophonological pattern-matching strategy while the P600 an automatic syntactic processing/repair mechanism. While HL proficiency supports syntactic reanalysis (P600), exposure and use enhance pattern-matching mechanisms (N400), strengthening early sensitivity to morphological regularities. Formal instruction further reinforces pattern-based processing, especially of less salient patterns while reversing markedness effect in grammatical processing.

## Figures and Tables

**Table 1.** Example grammaticality judgment task stimuli by condition

	Grammatical	Ungrammatical
Masculine noun	Mateo visitó un_masc_ pueblo_masc_ *pequeño*_masc_ con sus amigas. Mateo visited a_masc_ *small*_masc_ town_masc_ with his friends.	*Mateo visitó un_masc_ pueblo_masc_ *pequeña*_fem_ con sus amigas. *Mateo visited amasc *small*_fem_ town_masc_ with his friends.
Feminine noun	Leonor vió una_fem_ película_fem_ *romántica*_fem_ en el cine. Leonor watched a_fem_ *romantic*_fem_ movie_fem_ in the theater.	*Leonor vió una_fem_ película_fem_ *romántico*_masc_ en el cine. Leonor watched a_fem_ *romantic*_masc_ movie_fem_ in the theater.

## Data Availability

Supplementary materials and the data that support the findings of this study are openly available in the OSF at https://osf.io/ua3fb/?view_only=7138ca8f84e64d0fb519fcbe566fff15.
